# Effects of Sodium Chloride-Rich Mineral Water on Intestinal Epithelium. Experimental Study

**DOI:** 10.3390/ijerph18063261

**Published:** 2021-03-22

**Authors:** Pascual-Vicente Crespo, Fernando Campos, Manuel Leal, Francisco Maraver

**Affiliations:** 1 Tissue Engineering Group, Department of Histology, University of Granada, 18016 Granada, Spain; pvcrespo@ugr.es (P.-V.C.); fcampos@ugr.es (F.C.); 2 Professional School of Medical Hydrology, Complutense University of Madrid, Plaza Ramón y Cajal s/n, 28040 Madrid, Spain; hidromed@ucm.es; 3 Medical Hydrology Group, Department of Radiology, Rehabilitation & Physiotherapy, Complutense University of Madrid, Plaza Ramón y Cajal s/n, 28040 Madrid, Spain

**Keywords:** balneology, sodium chloride mineral water, hydropinic therapy, drinking cure, intestinal epithelium, osmotic effect, scanning electron microscopy, rats, animal study

## Abstract

Since knowledge concerning the cellular and tissue substrate that explains the therapeutic action of mineral waters is generally very scarce, we address the different effects that Lanjarón-Capuchina mineral water exerts on the intestinal epithelium in an experimental model as a prototype of the sodium chloride-rich mineral waters used in digestive disorders. In the experimental protocol, two groups of five adult Wistar rats received unrestricted mineral water in their diet or mineral water directly into the gastrointestinal tract through a catheter. A third control group was given a standard diet and water ad libitum. Intestinal samples for scanning electron microscopy were analyzed according to standardized methods. The observations carried out by microscope after the administration of the sodium chloride-rich mineral water clearly indicate that the hypertonic action of this mineral water affects the structure of the intestinal epithelium. It modifies the microvilli absorption in terms of the groups of enterocytes and the secretion of goblet cells, but it particularly affects the epithelial renewal process, accelerating and stimulating cell extrusion. The type of extrusion mechanism observed by microscope allows us to affirm that, although this increased after direct administration, it does not generate an epithelial disruption as it occurs in other circumstances with other extrusion modalities.

## 1. Introduction

Since ancient times, mineral waters have been used as curative agents for digestive disorders. Currently, the main indications are for dyspepsia, irritable bowel syndrome and constipation [1]. Among existing balneology studies examining the use of mineral waters in digestive disorders, we should highlight those carried out in: Helwan [2], Egypt; Contrexéville [3,4], France; Chianciano [5,6,7,8], Montecatini [9,10,11], and Uliveto [12], Italy; Nagayu [13], Japan; Rogaška Slatina [14], Slovenia; and Bursa [15], Turkey.

In Spain, in the natural region of Alpujarra, located in the south of the province of Granada, is the Lanjarón Spa. Its hydrogeological conditions allow the existence of a set of springs with unique characteristics. Among them is the spring of Lanjarón Capuchina dedicated to disorders of the digestive system [16]. For more than two hundred years, the waters of this spring have been applied orally in the treatment of functional disorders of the intestine, mainly in constipation, behaving due to its high mineralization as an osmotic purgative. Currently more than a thousand patients a year use them with good results.

Although the therapeutic activity of mineral waters, including those mentioned above, is closely related to the composition and concentration of mineral salts and to the different treatment guidelines applied, knowledge concerning the cellular and tissue substrate that explains the therapeutic action of mineral waters is very scarce [17,18,19]. Experimental studies in this field do not usually address this structural approach either [20,21].

In addition, an evaluation of the cellular and tissue bases which are underlying the therapeutic action of mineral waters is of interest, not only to better know the effects on that level of the biological organization, but also to correlate them with the physiological or physiopathological effects generated by mineral waters.

In the present study, we address the different effects that Lanjarón-Capuchina mineral water (L-CMW), as a prototype of sodium chloride-rich mineral water used for digestive disorders, has on the intestinal barrier in an experimental model. The high total dissolved solids (TDS) value of the mineral water employed in this study, regarding the rest of the sodium chloride-rich mineral waters, may help us to establish, a useful general model to explain the morphostructural effects generated by these types of waters on the intestinal surface. Our objective of study is the intestinal villi and especially the enterocytes that cover their surface, the main structures responsible for intestinal absorption. For this study, we selected scanning electron microscopy as the most relevant tool to identify the microscopic surface patterns both in studies applied to medical sciences and natural sciences [22,23,24].

## 2. Materials and Methods

### 2.1. Lanjarón-Capuchina Mineral Water

Strong mineralization, it contains: sodium, calcium, chloride, iron, and carbon dioxide. Their characteristics, described in Table 1, were determined in our laboratory by standardized methods: conductivity (2510 B), hardness (2340 b), Fe total (3111 B), CO_2_ (4500 CO_2_C), and Cl^−^, F^−^, Br^−^, NO_3_^−^, NO_2_^−^, and SO_4_^2−^ (4110 B), following Standard Methods [25]; Na^+^, K^+^, Li^+^, Ca^2+^, and Mg^2+^ (3007), following the Environmental Protection Agency [26], and HCO_3_^−^ (33017) following the Association of Official Analytical Chemists [27].

### 2.2. Experimental Protocol

**Figure 1:**
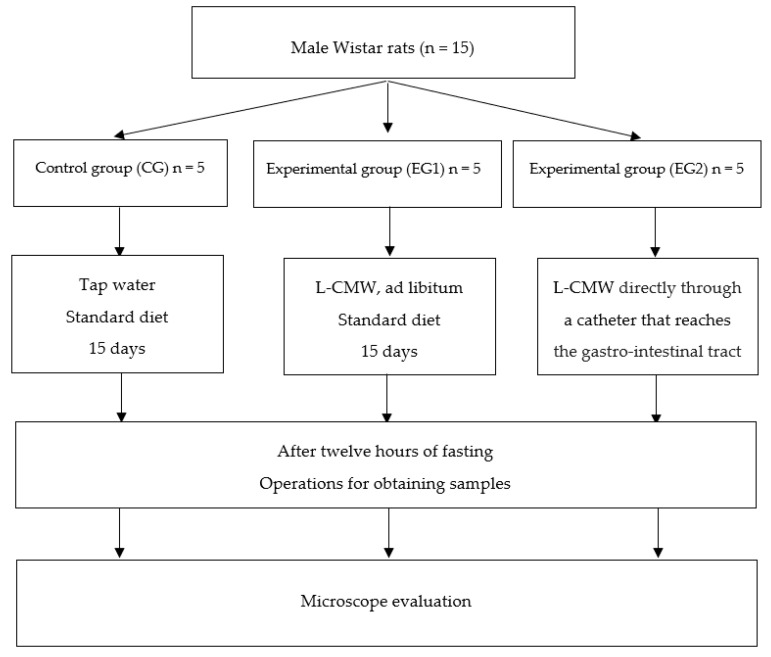
Animals and experimental design flow chart. L-CMW = Lanjarón-Capuchina mineral water.

Fifteen, 12-week-old male Wistar rats weighing 250–300 g were used to implement the experimental protocol. The animals were divided into three groups of five rats each. The animal distribution was at random and auxiliary variables such as sex and weight were previously taken into account to confer minimal variations to the groups according to the literature [29,30]. In the first group used as control (CG), the diet was standard laboratory food (SAFE^®^ A04, Augy, France) and water (tap water) ad libitum. The second group -Experimental group 1 (EG1)- also received a standard laboratory food (SAFE^®^ A04, Augy, France) and L-CMW, without restriction. The protocol was carried out in both groups for fifteen days according to the experimental protocol for this type of water [31]. In the third group -Experimental group 2 (EG2)- the rats received L-CMW (Lanjarón-Capuchina mineral water) directly through a gastric catheter that reaches the gastro-intestinal tract. Thirty cubic centimeters of mineral-medicinal water were introduced through this mechanism for ten minutes only once (Figure 1). Animals whose follow-up was carried out for fifteen days (CG and EG1) were caged in the animal facility in a temperature-controlled room (21 ± 1 °C) on a 12 h light/dark cycle. The operations for obtaining samples of all animals were performed, after 12 h of fasting, under an anesthesia cocktail of ketamine (30 mg/kg), xylazine (6 mg/kg), and acepromazine (1 mg/kg). After obtaining samples in live anesthetized animals, in order to avoid the rapid postmortem changes that take place in the intestinal tract, the animals were sacrificed. All experiments were carried out according to the European Union and Spanish Government guidelines for the ethical care of animals (EU Directive No. 63/2010, RD 53/2013). The study was approved by the ethics committee of the department of Physical Medicine and Rehabilitation. Medical Hydrology of Madrid Complutense University.

### 2.3. Microscopic Evaluation

The intestinal mucosa samples for scanning electron microscopy were fixed with 3% glutaraldehyde (Sigma-Aldrich, Steinheim, Germany) buffered with cacodylate (Sigma-Aldrich, Steinheim, Germany) at a temperature of 4 °C. The samples remained in the fixative solution for a minimum of 12 h and a maximum of 24 h. After this time, we performed three washes in cacodylate buffer for 10 min each, to remove the fixative (3% Glutaraldehyde) used previously. The samples were then postfixed in 1% osmium tetroxide (Sigma-Aldrich, Steinheim, Germany) for 90 min. After fixation, the samples were dehydrated using increasing concentrations of acetone (30%, 50%, 70%, 95%, and 100%) (Panreac, Barcelona, Spain), critical-point dried, whole-mounted on aluminium stubs under a magnifying glass for correct orientation of samples and, finally, gold sputter-coated according to routine procedures previously established [32,33]. Thirty samples, ten from each group, were analyzed in a scanning electron microscope using a voltage of 20 Kv (SEM Philips 505, Eindhoven, The Netherlands). Of the ten samples analyzed in each group, five belong to the duodenum and five to the ileum. After processing the samples according to the methodology indicated above, each one was independently observed by two researchers and the characteristics corresponding to the type of microvilli, microvilli pattern of enterocytes, secretory pattern of goblet cells, and extrusion patterns on the tip of the villi were recorded.

### 2.4. Statistical Analysis

SEM images from enterocytes surface were selected and processed in the threshold function by using Image J software (National Institutes of Health, Bethesda, MD, USA), as previously described [34], in order to quantify intestinal microvilli. SPSS software version 24.00 (SPSS Inc., Chicago, IL, USA) was used to determine significant differences between the comparisons of the different samples studied. A Mann–Whitney non-parametric test was used and *p*-values < 0.05 were considered statistically significant.

## 3. Results

In this section, we will describe firstly the three-dimensional pattern of the intestinal villi and secondly the microscopic pattern of the cell surfaces of the intestinal epithelium. This was performed in the three experimental groups.

### 3.1. Intestinal Villi

**Figure 2:**
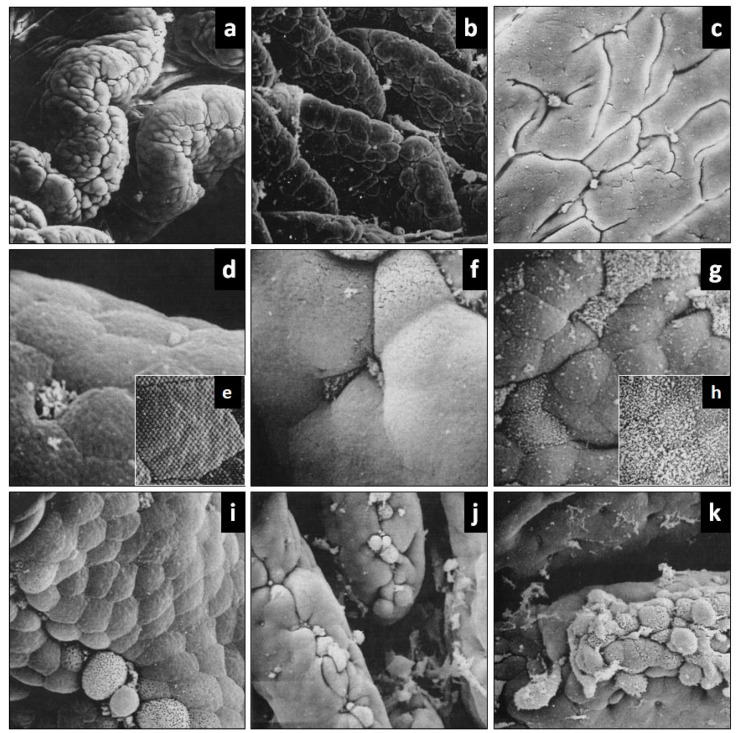
Scanning electron microscopy images of the intestinal surface in the control group and after administration of L-CMW in the two experimental groups: (**a**) CG. Type I intestinal villi. ×320; (**b**) CG. Type II intestinal villi. ×180; (**c**) EG2. Folds in the side wall of type I villi with cerebroid appearance. ×600; (**d**) CG. Enterocytes and goblet cells. ×3100; (**e**) CG. Distinctive microvillous pattern of the apical surface of the enterocyte. ×12,000; (**f**) EG1.Enterocytes and goblet cells. ×3.100; (**g**) EG2. Areas of enterocytes with different patterns of microvilli on their surface. ×2200; (**h**) EG2. Loss of the distinctive morphological pattern of enterocytes. ×5.200; (**i**) CG. Cellular extrusion process at the tip of the villi. ×1.500; (**j**) EG1.Cellular extrusion process. ×750; (**k**) EG2. Cellular extrusion process. ×960.

Two types of villi, types I and II, were observed in the duodenum and ileum of the control and experimental animals studied. Type I villi are predominantly observed in the duodenal region of the intestine and are mainly characterized by presenting a circumvallate or S italic morphology (Figure 2a). Type II villi haves a foliaceous or leaf-shaped morphology (Figure 2b). This villous type has been frequently observed in the ileal region. Although the effects of L-CMW modified the two types of villous patterns, especially at the level of the enterocytes, only group EG2 showed folds with a cerebroid appearance in relation to group EG1 and the control group where the changes are less relevant (Figure 2c).

### 3.2. Enterocytes and Goblet Cells

Regarding the enterocytes and goblet cells, an evolution is observed from the orthotypical pattern of the control group to the heterotypical patterns present in both experimental groups for the main cells of the intestinal epithelium, especially in the group in which L-CMW was applied directly to the intestinal surface (Figure 2d–h).

The flattened surfaces of the enterocytes of the control group present a distinctive hexagonal pattern with a compact organization of the microvilli occupying almost all the surface (Figure 2d,e).The mean and standard deviation of the microvilli on the enterocyte cell surface is 2083 ± 860.3. However, when L-CMW is administered in the diet or directly, the surface of some groups of enterocytes becomes cupuliform, the hexagonal pattern is altered and the microvilli lose their compactness, having a flexible appearance (Figure 2f). These phenomena increase when the medicinal water is applied directly, observing microvilli of different sizes and even small areas of enterocytic surface devoid of microvilli (Figure 2g,h). The mean and standard deviation of the microvilli on the enterocyte cell surface is 262 ± 115.2. There are statistically significant differences between the CG and EG2 groups regarding the number of microvilli on the enterocyte cell surface (*p* < 0.021). Remains of granular material are observed in both experimental groups on the apical surface of these cellular elements.

Open pole goblet cells that are located at the level of the intestinal epithelium are relatively rare in type I intestinal villi, but their presence increases in type II villi. These cellular elements were observed with scanning electron microscopy as small depressions of variable size, quite scattered along the wall of the intestinal villi, and very rarely in the apical region of the villi (Figure 2a,b,d). The identification of goblet cells in the epithelium of the intestinal surface is possible due to the presence in these depressions of well-defined spheroidal formations of variable size constituted by a mucoid material, a product of cellular secretion.

After administration of L-CMW in the diet or directly through a catheter onto the intestinal surface, the goblet cells show, by scanning electron microscopy, the same morphostructural characteristics previously described in the control group. However, in both cases, the increase in the size of the depression on the surface, that characterizes its presence, is very marked and sometimes reaches the size of the surface of the enterocytes (Figure 2d). It is common to observe, on the apical surface of these goblet cells, bulky mucoid globular material, also a product of an increased secretory activity. When medicinal water is applied directly, it is also common to observe areas of mucus on the intestinal epithelium, which sometimes makes microscopic observation difficult.

### 3.3. Cell Extrusion Process

A remarkable fact, observed in the three groups studied, is the cellular extrusion process that takes place at the tip of the villi. Along the apical border, groups of generally spheroidal cells with scattered microvilli are being extruded and desquamated in the final process of renewal of the intestinal epithelium (Figure 2i). While this process is limited in the control group, the cells show a fairly homogeneous volume in the experimental groups, and especially the group in which the medicinal water is applied directly, the groups of cells are more numerous and heterogeneous with cells of very diverse volume and surface patterns (Figure 2j,k).

### 3.4. Other Cells

In addition to the enterocytes and goblet cells identified in our study other cell types, whose percentages are extremely low can also be observed on the surface of the intestinal epithelium with scanning electron microscopy. These cells are M cells and the caveolate cells that do not present significant modifications compared to the control samples.

## 4. Discussion

At present, treatments using mineral waters with a TDS greater than 1 g/L are used in balneology for digestive disorders, especially for dyspepsia [8,9,10,11,12,31,35,36,37,38], bowel function alteration and constipation [3,4,14,15,35,36,37,39,40,41,42], biliary tract dysfunctions [5,6,7,43], metabolic syndrome [44,45,46,47,48,49], and others [13,50,51,52,53].

However, no water used in the literature cited has as high a TDS as L-CMW “18,221 mg/L” [28]. This allows this mineral water to act as an osmotic laxative, which, depending on the dose and individual tolerance, has a choleretic, cholagogue and laxative or purgative effect.

On the other hand, there are more and more studies that use animals to explain the mechanisms of action of balneology [20,21], for example, those carried out in bone and joint pathology [54,55,56,57], metabolic syndrome [2,58,59,60,61] and others [12,62,63]. However, as indicated above, these investigations do not address the structural substrate that supports the action of these waters. In this sense, there are no studies that highlights the structure of the intestinal surface after the use of mineral waters to treat digestive disorders.

In the present study we have used L-CMN, with the characteristics indicated above, in an experimental model in which the action of the mineral water is exerted after its administration in the diet or after direct administration on the intestinal surface through a catheter. Regarding the effect of anesthesia and drugs used in our protocol, it is important to note that ketamine-acepromazine-xylazine has long been a popular combination of injectable anesthetics recommended for use in laboratory rodents. In the present work, its administration was also selected not only because its side effects do not generate abdominal lesions as results of anesthesia but also because it does not generate significant alteration in the release of relevant cytokines such as TNF-alpha [64,65,66].

The microscopic observations carried out with scanning electron microscopy, which allows a three-dimensional observation of biological surfaces, highlights the existence of a morphostructural microscopic pattern compatible with the alteration of cell volume homeostasis and ionic changes generated by the action of this type of mineral water, not only on other biological surfaces but also in isolated cell models [19,67]. In addition, these microscopic patterns show a significant degree of variation in relation to the experimental administration of mineral water.

The intestinal surface is a specialized epithelial barrier that separates the intestinal lumen from the inner compartments of the body. Epithelial cells have an important role in this barrier concerning the movement of solutes from one side to the other. As water follows the movement of the solutes, any disruption in the cell membrane will conduce to significantly disrupt cell volume [19]. When the intestinal epithelial cells are exposed to a hypertonic solution, as occurs after the administration of L-CMW, they first lose water to reach a balance between the internal and external osmotic pressures. After this initial reduction in cell volume, some cells display a regulatory volume increase (RVI) process to recover their volume. However, other epithelial cells do not display that regulatory mechanism and show different degrees of shrinkage. Our results showed that L-CWM does not alter the villi patterns of the intestine because these patterns are solidly supported by the connective axes of the lamina propria [68]. The cerebroid appearance of the lateral walls of the villi and the alterations observed in groups of enterocytes in both experimental groups, especially concerning their surface morphology and microvillus pattern, are compatible with the regulatory cell volume phenomena previously indicated. The superficial expansion of the secretory pole of goblet cells, may be observed using scanning microscopy as a wide depression existing between the enterocytes, which would indicate that these cells are also affected by the regulatory process of cell volume after the hypertonicity generated by the administration of L-CMW. Regarding the ions involved in the process, the recovery of volume through the RVI, after the acute shrinkage caused by a hypertonic medium, is mediated, as pointed out by Carbajo and Maraver, by keratinocytes, with the intracellular accumulation of salts (predominantly NaCl and KCl) and by the water carried by these electrolytes through the cellular exchange of hydrogen with sodium (Na^+^/H^+^) and chlorides and bicarbonates (Cl^−^/HCO_3_^−^) that regulate the pH or through the channels of Na^+^/K^+^/2Cl co-transporter and Na^+^ [19].

These changes in the cellular volume of the intestinal epithelial barrier also have other consequences that are microscopically evident in our study in which statistically significant differences are observed between the microvilli pattern of the enterocytes in the control group and the enterocytes subjected to the direct action of L-CMW. In addition, it has been described that the cell volume plays a very important role in different cellular functions such as growth, differentiation, migration, and cell death [19,67,69]. Microscopic observation of the intestinal surface after the administration of L-CMW, through the diet or directly through a catheter, highlights that most of these phenomena are significantly activated. In fact, cell growth, differentiation, migration, and apoptosis are evidenced in the important process of cellular extrusion that is observed at the tip of the villi and that is much more intense after the direct administration of mineral water.

In normal intestinal epithelium, the balance between the apoptosis and extrusion processes of the epithelial cells at the tip of the villi, and the generation of new cells in the epithelial crypt, is essential to maintain the homeostasis of the epithelial barrier [70]. The effect of L-CMW during both types of administration used in our experimental model alters the homeostatic balance in the epithelial barrier. It has been well documented that variations in cell volume, associated with osmotic variations and ion exchange, play an important role in the process of cell division, differentiation, migration and apoptosis, all of which are phenomena involved in the normal renewal of the intestinal epithelium [67,70,71]. As described in the results section, cell extrusion is present at the tip of the villi with more numerous cell groups and more heterogeneous patterns in both experimental groups, especially in the one that receives the mineral water directly. It is also relevant to point out the modifications to the patterns observed in the extrusion process and its increase when L-CMW mineral water is applied directly to the intestinal surface. Epithelial cell apoptosis, which supports this mechanism, is significantly triggered when the cells are exposed to hypertonic cell shrinkage [67]. Therefore, the ability to regulate cell volume by some groups of cells determines their greater or lower resistance to apoptosis and, consequently, their participation in accelerating the process of epithelial renewal and in the heterogeneity of the patterns observed at the tip of the villi.

Although three types of extrusion have been described under different circumstances [72], the process and mechanism of extrusion observed in our study is compatible with extrusion mode 1, according to which the enterocytes, at the tip of the villi, are detached from the basal lamina and ascend, within the epithelium, modifying their union systems with the neighboring enterocytes. After passing through a “polyp-like” phase, in which the enterocyte remains attached to the epithelium by a thin band of cytoplasm located among other enterocytes, the cell is finally detached from the intestinal lumen [70,72]. This process is more evident and presents a more heterogeneous aspect on the cell surface with the use of mineral water, especially when it is applied directly on the intestinal surface. No morphostructural pattern corresponding to cell extrusion types 2 and 3, characterized by the extrusion of apical cell fragments or scattered holes on the surface of villi corresponding to spaces of detached enterocytes and consequent epithelial disruption, were observed.

On the other hand, the results obtained in this experimental study justify the clinical benefits observed over seventeen years by one of the authors of the paper, as a doctor at the Lanjarón medical spa. In fact, the treatment with L-CMW is associated with an improvement in the intestinal function, which lasts up to three months after the end of the drinking cure and to a lower consumption of laxatives [16].

The results we have described and interpreted in the context of the existing literature have, however, some limitations. Firstly, most studies on cellular volumetric changes in relation to hypotonia and hypertonia have been carried out using isolated cellular models such as Ehrlich’s ascites tumor cell model, but not in vivo epithelial models affected by such variations [19,67]. This involves the need to extrapolate the experimental results observed to those referred to in such models. On the other hand, it is important to note that although there is fibrillar-mucoid material of a secretory origin on the cell surface in the microscopic observations, the sample preparation techniques for scanning electron microscopy largely eliminate such secretory material deposited on the surface. Because of this reason, it is not possible to correlate the material observed with the secretory activity of the goblet cells on the intestinal surface appropriately. However, it is important to point out that this circumstance does not prevent the possibility of identifying the surface patterns described in this study after the use of L-CMW clearly. Finally, a limitation of our study is the use of a single type of mineral water that is only justified by its peculiar chemical characteristics described in Table 1 and its therapeutic applications [16,28]. Further experimental biochemical and functional studies in vivo regarding the effects of L-CMW in the intestinal epithelium of rats could better correlate the microscopic patterns described for the first time in this study with the different therapeutic actions postulated for this mineral water.

## 5. Conclusions

In the study carried out after the administration of L-CMV, using a mineral water with a high level of TDS, the modification of the microscopic pattern of absorption of microvilli from enterocytes and of secretion of the goblet cells should be highlighted. In addition, the direct action of L-CMW on the intestinal epithelium generates a process of cell extrusion of a type I pattern that reveals an accelerated process of epithelial renewal. The type 1 extrusion mechanism observed in the microscopic study carried out allows us to affirm that, although it is greatly increased when water is administered directly, it does not generate epithelial disruption as occurs in other extrusion modalities described in other circumstances. The administration of L-CMW associated with the diet achieves, intermediate effects between those observed in the control group and those associated with the direct administration of L-CMW by catheter. Clinical studies in the future should not only take advantage of the therapeutic potential of the action of this mineral water, but also establish the appropriate application of both routes of administration, i.e., together with the diet or by direct administration, in relation to its treatment of different digestive disorders.

## Figures and Tables

**Table 1:** Water analysis [28].

Source Name	Lanjarón-Capuchina		
Type of Water	Strong Mineralization. Sodium, Calcium, Chloride, Iron, Carbon Dioxide		
Physicochemical properties			
Temperature	21.1 °C		
Conductivity to 25 °C	32,590 μS.cm^−^^1^		
pH value	6.04		
TDS to 180 °C	18,221 mg/L		
Alkalinity	90.25 mg/L CaCO_3_		
Anions	mg/L	mEq/L	% mEq
Cl^−^	9011.6	254.216	86.23
F^−^	0.8	0.044	0.01
HCO_3_^−^	1791.0	29.354	9.96
Br^−^	22.9	0.286	0.10
NO_3_^−^	21.2	0.341	0.12
NO_2_^−^	1.3	0.027	0.01
SO_4_^2^^−^	506.7	10.549	3.57
Total	11,355.4	294.819	100.00
Cations	mg/L	mEq/L	% mEq
Na^+^	4054.1	176.355	59.50
K^+^	542.4	14.092	4.75
Li^+^	25.2	3.628	1.22
Ca^2+^	1637.8	81.727	27.57
Mg^2+^	238.0	19.582	6.61
Fe total	28.5	1.021	0.35
Total	6526.0	296.404	100.00
Gas			
CO_2_ dissolved (mg/L)	342.6		
H_2_S dissolved (mg/L)	0.0		
